# Novel protocol for multiple-dose oral administration of the L-type Ca^2+^ channel blocker isradipine in mice: A dose-finding pharmacokinetic study

**DOI:** 10.1080/19336950.2024.2335469

**Published:** 2024-04-02

**Authors:** Tamara Theiner, Nadine J. Ortner, Herbert Oberacher, Gospava Stojanovic, Petronel Tuluc, Jörg Striessnig

**Affiliations:** a Department of Pharmacology and Toxicology, Institute of Pharmacy, Center for Molecular Biosciences Innsbruck, University of Innsbruck, Innsbruck, Austria; b Institute of Legal Medicine and Core Facility Metabolomics, Medical University of Innsbruck, Innsbruck, Austria

**Keywords:** Calcium channel blockers, dihydropyridines, pharmacokinetics, drug administration, voltage-gated calcium channels

## Abstract

Studies in genetically modified animals and human genetics have recently provided new insight into the role of voltage-gated L-type Ca^2+^ channels in human disease. Therefore, the inhibition of L-type Ca^2+^ channels in vivo in wildtype and mutant mice by potent dihydropyridine (DHP) Ca^2+^ channel blockers serves as an important pharmacological tool. These drugs have a short plasma half-life in humans and especially in rodents and show high first-pass metabolism upon oral application. In the vast majority of in vivo studies, they have therefore been delivered through parenteral routes, mostly subcutaneously or intraperitoneally. High peak plasma concentrations of DHPs cause side effects, evident as DHP-induced aversive behaviors confounding the interpretation of behavioral readouts. Nevertheless, pharmacokinetic data measuring the exposure achieved with these applications are sparse. Moreover, parenteral injections require animal handling and can be associated with pain, discomfort and stress which could influence a variety of physiological processes, behavioral and other functional readouts. Here, we describe a noninvasive oral application of the DHP isradipine by training mice to quickly consume small volumes of flavored yogurt that can serve as drug vehicle. This procedure does not require animal handling, allows repeated drug application over several days and reproducibly achieves peak plasma concentrations over a wide range previously shown to be well-tolerated in humans. This protocol should facilitate ongoing nonclinical studies in mice exploring new indications for DHP Ca^2+^ channel blockers.

## Introduction

Voltage-gated L-type Ca^2+^-channels in the cardiovascular system are an important drug target. L-type Ca^2+^-channel blockers (CCBs) are well-established for the evidence-based treatment of hypertension and angina. Unlike verapamil and diltiazem, dihydropyridine (DHP) CCBs – such as amlodipine, felodipine, and isradipine (ISR) – lack cardiodepressant effects at therapeutic doses. They are widely used, well-tolerated and safe. Their vasodilatory actions are mediated by the Cav1.2 isoform [1], one of the four L-type Ca^2+^-channels (Cav1.1-Cav1.4 [2]). Due to alternative splicing and the voltage-dependent mode of DHP action, inhibition of Ca1.2 channels in the working myocardium requires much higher plasma concentrations (C_PL_) than in smooth muscle [1,3]. Cav1.2 channels support physiological functions in a wide variety of electrically excitable cells, including neurons and endocrine cells. In many cells, such as sinoatrial node cells, neurons, chromaffin cells and pancreatic islet cells, Cav1.3 channels also contribute to L-type currents. Their gating properties differ from Cav1.2 allowing them to support functions distinct from Cav1.2 [1].

DHPs are widely used as pharmacological tools to study Cav1.2 and Cav1.3 functions outside the cardiovascular system and their implication in the pathophysiology of a variety of human diseases, including drug addiction, neuropsychiatric disorders, Parkinson’s Disease, and spasticity after spinal injury [1,4–6]. In animal models of human diseases mostly acute DHP effects on behavioral or other disease-relevant readouts are investigated. These drugs have a short plasma half-life especially in rodents [7], and show high first-pass metabolism upon oral application. They have therefore been mostly delivered through parenteral routes *in vivo*, either directly, or through osmotic minipumps or subcutaneous drug-releasing pellets (e.g [8–12]). Pharmacokinetic data measuring the C_PL_ achieved with these applications are sparse [8,10,12,13]. However, this is essential because high doses of DHPs cause side effects, evident as DHP-induced aversive behaviors confounding the interpretation of behavioral studies [14,15]. Moreover, injections and subcutaneous implants require animal handling and can be associated with pain, discomfort, and stress which could influence a variety of physiological processes, behavioral, and other functional readouts. Therefore, a noninvasive application of DHPs not requiring animal handling but resulting in predictable C_PL_ is needed. We were therefore aiming at a protocol allowing fast, stress-free and reliable oral dosing to produce predictable C_PL_ for several hours in mice to allow the study of acute and early subacute effects of ISR within a predefined dose range.

Here, we report a dose-finding pharmacokinetic study for the oral application of the CCB ISR in mice. Drug application is based on voluntary consumption, is stress-free, and can reproducibly achieve C_PL_ within a wide range previously shown to be well-tolerated in humans.

## Methods

All animal experiments were approved by the Austrian Animal Experimentation Ethics Board (BMWFW-66.008/0008-WF/II/3b/2014, BMWFW-66.008/0008-WF/V/3b/2017, BMWFW-66.008/0019-WF/V/3b/2017).

### Oral in vivo isradipine (ISR) administration

**Figure 1.**
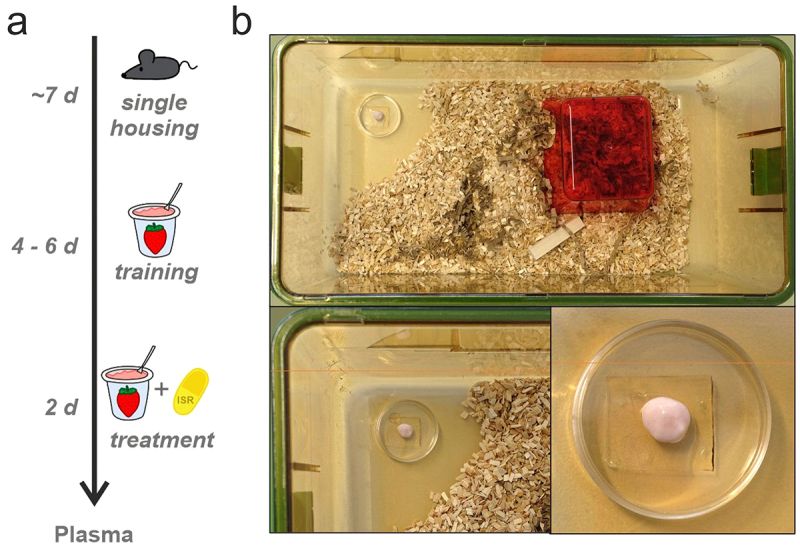
Illustration of the experimental setup for voluntary oral ISR administration. (a) Schematic overview of the experimental design. Mice were single housed for approximately one week, followed by the training period with drug-free strawberry-flavored yogurt (SFY) for 4 to 6 days. Subsequently, mice were treated with multiple-doses for two consecutive days with a specific ISR dose (as indicated for each cohort) mixed into SFY immediately before administration. (b) Mouse cage with standard housing conditions (top). In the front part of the cage, SFY with or without ISR is presented in a 35 mm cell culture dish, which is glued to the cage floor with bedding material removed around it (shown in enlargements at the bottom).

Male and female WT mice with a C57BL/6N genetic background (age: 11–18 wk) were single-housed for at least 1 week prior to ISR administration (Figure 1a). Mice were kept under standard housing conditions with controlled temperature (22°C) on a 12 h light/dark cycle with food and water available ad libitum. Each cage was provided with standard bedding and nesting material, shelters, and gnawing sticks (Figure 1b).

Before the start of drug treatment mice were trained for 4–6 days to voluntarily consume strawberry-flavored yogurt (SFY) (“Fruchtzwerge,” Danone, Germany; contains 6.7% protein, 11.4% carbohydrate [of which 10.6% sugar], 2.6% fat, 97 kcal/100 g, 80 mg salt/100 g), which was used as a vehicle for the administration of ISR (Figure 1a). In the training phase mice received 200–500 µl drug-free SFY 2–3 times a day on a 35 mm cell-culture dish placed at the front part of the cage floor (Figure 1b). At the end of the training period the majority of the mice finished to eat the SFY within 1–5 minutes after presentation. The training phase also allows mice to habituate to the feeding procedure, thus minimizing novelty responses caused by SFY/drug presentation. After the training period oral ISR administration started by providing them with 0.05–0.5 mg ISR mixed into 200–500 µl of SFY per feeding in single- or multiple-dose studies with different dosing intervals in five independent cohorts. ISR was provided as an extended-release formulation contained in Vascal® uno 5 mg capsules (Cheplapharm Arzneimittel GmbH, Greifswald, Germany). Capsules were opened and the extended-release matrix granules were added to the SFY to obtain the indicated target doses (freshly prepared immediately before administration). Animals were observed during application and the application time was recorded together with any unusual behaviors (Table 1 and Supplementary Table S1). ISR plasma levels were determined in blood samples at preplanned time points after the mice finished their dose to assess pharmacokinetic parameters. The exact time period between finishing all SFY of the last dose and blood sampling was determined and was used for plotting and analyzing the data. More details of ISR administration and collection of blood samples from sacrificed mice are described in Supplementary Methods and in Table 1. Since no significant differences between male and female mice were observed, data was pooled for analysis. Data from cohort 5 has been used in a recent study investigating ISR effects on locomotion [16].

**Table 1.** Pharmacokinetic parameters after oral ISR administration. For each cohort we used a different ISR dosage and a different dose interval. The remaining ISR plasma concentrations (C_PL_) after certain time points are indicated as C_PL_ and their ln-transformed values (lnC_PL_). Data are presented as mean ± SD, with N-numbers indicated in the table. An estimate of the half-life (t½) was calculated for each cohort from ln-transformed data points as described in the Supplementary Methods.

Cohort	Dose *(mg)*	Time *(h)*	C_PL_ *(ng/ml)*	lnC_PL_ *(ng/ml)*	N	t½ *(h)*
1	0.5	1	56.2 ± 11.78	4.02 ± 0.21	3	
		4	39.4 ± 17.31	3.61 ± 0.42	3	1.35
		12	0.63 ± 0.28	−0.53 ± 0.37	5	
2	0.1	1	14.9 ± 5.27	2.64 ± 0.44	4	
		4	2.36 ± 0.73	0.83 ± 0.28	4	1.49
		8	0.49 ± 0.54	−1.06 ± 0.81	5	
3	0.05	0.5	3.03 ± 0.70	1.09 ± 0.23	4	
		2	3.03 ± 0.67	1.09 ± 0.21	5	0.81
		4	0.56 ± 0.19	−0.63 ± 0.41	4	
4	0.1	4	7.42 ± 2.04		3	
5	0.1	2	14.1 ± 8.09		9	

### Plasma isolation

Mice were sacrificed by cervical dislocation after sedation with isoflurane. Mice were decapitated and 0.5–1 ml of trunk blood were immediately collected in 1 ml MiniCollect tubes (Greiner bio-one 450,531, containing EDTA as an anticoagulant). Samples were centrifuged at 4°C for 10 min at 3,000 rpm (845 × g) and plasma immediately stored at −20°C.

### Plasma ISR measurements

Details are provided in the Supplementary Methods. ISR concentrations were determined as previously described [12,16] by liquid chromatography-tandem mass spectrometry (LC-MS/MS). An isotopically labeled analog was used as internal standard (ISR-d3). Sample preparation involved a protein precipitation step. Before use, plasma samples were thawed and allowed to equilibrate to room temperature. A 50 µl-aliquot of the sample was then mixed with 5 µl of internal standard solution (30 ng/ml) and 95 µl of methanol. The mixture was vortexed and centrifuged at 4,600 × g for 5 min. The obtained supernatant was submitted to LC-MS/MS analysis. Calibration was accomplished by complementing ISR-free mouse plasma with known concentrations of ISR ranging from 0.020 to 100 ng/ml. The limit of quantification was 0.050 ng/ml.

### Assessment of safety and tolerability

Mice were observed and assessed for any SFY and/or ISR-induced adverse effects, including abnormal behaviors before and during drug application, on a regular basis in between applications and before obtaining blood. The only abnormal symptoms newly observed after the start of treatment and consequently classified as adverse effects were any of the following (Supplementary Table 1): abnormal appearance with rough hair coat and piloerection, diarrhea, hunched posture, reduced yogurt intake, and reduced mobility. Symptoms were classified as “likely drug-related” if they appeared after ISR- but not during SFY-only treatment or if they were associated with abnormally high ISR C_PL_. All animals were included in the safety analysis. Mice consuming less than 75% of the last dose preceding plasma collection were excluded from the analysis of C_PL_ (indicated in Table 1).

## Results

In *in vivo* nonclinical studies, DHP CCBs are mostly delivered through parenteral routes. This requires animal handling or even surgery and can be associated with pain, discomfort, and stress which could affect behavioral and other functional readouts. Therefore, a noninvasive application of DHPs not requiring animal handling but resulting in predictable C_PL_ is needed. Although the addition of DHPs (including ISR) to chow has previously been used to treat rodents with DHPs, the onset of plasma levels is slow (5–10 hours to the maximal C_PL_, C_max_, in rats [13]) and the onset of drug action and the applied dose (calculated post-hoc) depends on food consumption.

The aim of this pharmacokinetic dose-finding study in mice was to develop an oral application procedure for the CCB ISR, which does not interfere with the usual daily behavior of mice, can be easily administered, is well tolerated and identifies oral doses leading to ISR C_PL_ achieved during the oral treatment with recommended therapeutic doses in humans of 1–11 ng/ml [17–22]; SPC Lomir^R^: compendium.ch/mpro/mnr/2604/html/). In addition, we also aimed at higher C_PL_ known to be tolerated by subjects in pharmacokinetic studies employing higher than approved oral doses or of unapproved oral formulations (peak plasma concentrations of up to 14.9–41.8 ng/ml [21,23]). This higher tolerable dose range is relevant to ongoing preclinical studies in mice exploring new indications for DHP CCBs requiring efficient brain penetration and/or engagement of L-type Ca^2+^ channel isoforms, which are slightly less sensitive to DHPs (such as Cav1.3) than currently targeted Cav1.2 channels in the cardiovascular system [12]. Since our intention was not to perform a detailed analysis of pharmacokinetic parameters, which can be already inferred from studies in rats [13], we used a minimal number of animals allowing solid conclusions regarding the C_PL_ time course of different oral doses and their inter-individual variability.

Vascal® uno is an extended-release formulation of ISR-containing matrix granules in a single capsule approved for human use in European countries at the time of this study. The powder could be easily mixed with different volumes of SFY and was then presented to the single-housed animals as shown in Figure 1. Note that animals had free access to their regular chow and water at all time (food basket not shown in Figure 1 for clarity).

During all training phases in the course of this study, SFY was well tolerated and no adverse events were observed.

In cohort 1 (*N* = 13, Supplementary Table S1) we first tested if the animals could be trained to voluntarily eat 500 µl SFY every 12 hours, the volume intended for the application of the highest ISR dose. On day 1, mice required between 1 and 4 hours to completely finish this amount of SFY, but the time was reduced to 15 min on day 2 and to 5 min on day 5 (13/13 mice). To test for multiple-dose tolerability and safety of a high dose of ISR first, we supplied 500 µl of SFY with 0.5 mg of ISR. This dose was selected because the animal equivalent dose for mice for an approved human therapeutic dose of 5–10 mg/60 kg (= 0.083–0.166 mg/kg) corresponds to 1.02–2.04 mg/kg (multiply human equivalent dose by 12.3 [24]), which corresponds to 0.02–0.04 mg for a 20 g mouse. We selected a slightly higher dose of 0.5 mg as the most likely highest tolerable oral dose in mice because i. ISR undergoes a prominent first-pass metabolism, which reduces oral bioavailability in rodents (F = 5% in rats [13]) even more than in humans (16–18%, SPC Lomir^R^: compendium.ch/mpro/mnr/2604/html/); ii. higher than approved single ISR doses (15 mg immediate release [22]) were well tolerated by human subjects; and iii. the body weight of our mice in cohort 1 was larger than 20 g (27.0 ± 3.19 g; Supplementary Table S1).

**Figure 2.**
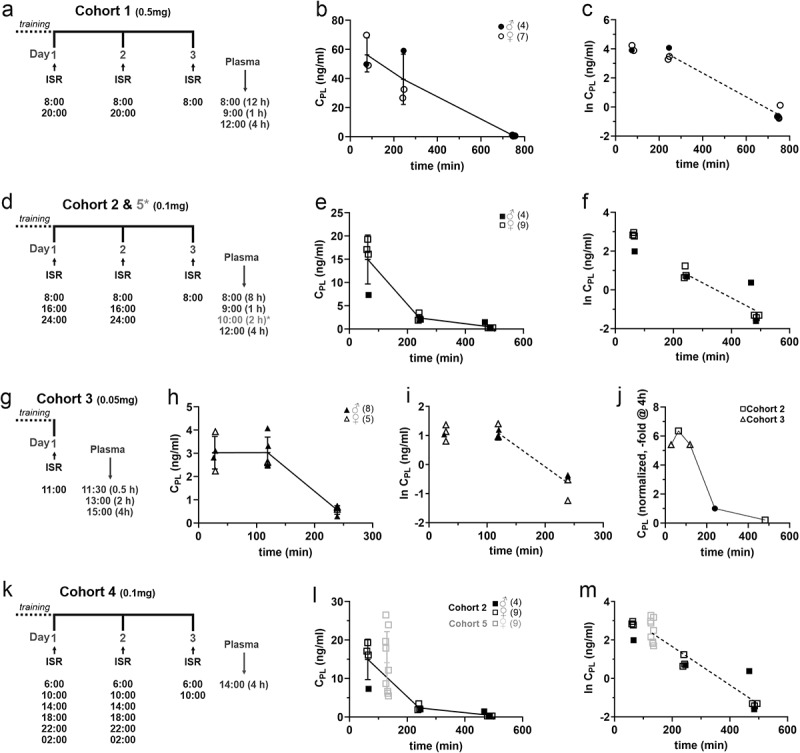
Pharmacokinetic analysis of orally administrated extended-release ISR in SFY. The experimental design for each cohort is shown on the left (a,d,g,k). (a) In cohort 1 mice were administrated 0.5 mg ISR in 500 µl SFY per feeding every 12 h (1 mg/day) at the indicated time points. On day 3, plasma was taken after 12 h, for which the mice received their last dose in the evening of the second day. Plasma was also collected 1 and 4 h after the morning dose on day 3. Two mice were excluded from analysis (see legend to Table 1). (b) the C_PL_ - time profile is illustrated with data shown separately for females (*N* = 7, open circles) and males (*N* = 4, filled circles). (c) Semi-logarithmic plot of the C_PL_-time course. (d-f) Corresponding protocol and data for cohort 2 in which mice (females: *N* = 9, open squares; males: *N* = 4, filled squares) were offered 0.1 mg ISR in 300 µl SFY per feeding every 8 hours (0.3 mg/day). The same protocol was used for cohort 5 but C_PL_ was only measured 2 h after drug application (*, data see panels l, m). (g-i) Corresponding protocol and data for cohort 3 in which mice (females: *N* = 5, open triangles; males: *N* = 8, closed triangles) after the training period mice received a single dose of 0.05 mg ISR in 200 µl SFY and plasma was collected after 30 min, 2 and 4 h. (j) Mean plasma levels obtained in cohorts 2 and 3 were normalized to their mean value at 4 h (filled circle), which allowed to combine their C_PL_ measured at different time points into one curve (open symbols). (k) Protocol for cohort 4. Mice (females, *N* = 3) were treated for two consecutive days every 4 h with 0.1 mg ISR in 200 µl SFY. On day 3, plasma was taken 4 h after the last ISR administration as an estimate for trough concentration to test for drug accumulation upon multiple dosing (see results). (l, m) In cohort 5 mice (*N* = 9 females, light grey) were treated like in cohort 2 but plasma was collected only 2 h after the last dose at 8:00 am on day 3. In panels l and m the C_PL_ data obtained after 2 h (open squares, light grey) are plotted into the C_PlPL_-time course of cohort 2 to illustrate that the mean C_Pl_ of cohort 5 falls into the range predicted from previous experiments (j), demonstrating the reproducibility of the regimen. All error bars represent ± SD.

In a multiple-dose setting, 0.5 mg ISR were offered to trained mice in 500 µl of SFY for 2 days every 12 hours (daily dose of 1 mg/d) and were then sacrificed 12 h after the last dose on the next day and after approx. 1 h and 4 h after an additional dose on day 3 (Figure 2a; Supplementary Table S1). 8/13 mice consumed the last dose completely and within 5–60 min, three finished only after more than 60 min. One mouse refused or partially refused consumption of 3 of the 5 doses presented and another one consumed only 25% of the last dose. Both were therefore excluded from C_PL_ analysis. Although this dose did not prevent most of the mice to finish the offered dose, less reliable consumption of ISR-SFY as compared to the SFY alone during the training phase already indicated reduced tolerability. Therefore, no higher doses were tested.

The linear (Figure 2b) and semi-logarithmic plot (Figure 2c) of the C_PL_-time course of the remaining mice (*N* = 11) is shown in Figure 2. The C_PL_ declined from a maximum of 56.2 ± 11.8 ng/ml (*N* = 3) at 60 min to a minimum of 0.63 ± 0.3 ng/ml (*N* = 5, Table 1) at 12 hours (which was well within our lower limit of detection, see methods). From the slope of the curve between 4 h and 12 h an apparent half-life of the C_PL_ decay of about 1.4 h could be estimated. The maximal C_PL_ observed is within the range of the highest C_PL_ measured in humans after oral application of a single 15 mg oral dose (41.8 ± 14.7 ng/ml, mean ± S.D [23]) and far below C_PL_ levels reached immediately after a single 1 mg i.v. dose (>100 ng/ml [21]).

To cover a lower C_PL_ range, closer to those reported at usual doses in humans (2 × 2.5–10 mg/d immediate release or 1 × 5–20 mg/d continuous release ISR), ranging from C_max_-values between 1 and 11 ng/ml ([17–21]; SPC Lomir^R^: compendium.ch/mpro/mnr/2604/html/), we reduced the dose to 0.1 mg in cohort 2 (Supplementary Table S1, Figure 2d-f). Due to the rapid elimination, we also reduced the dosing interval to 8 hours to increase the time with significant ISR exposure but without expecting cumulation during multiple dosing. Moreover, the volume of SFY for drug delivery was also further reduced to 300 µl (Supplementary Table S1). Animals were trained to consume SFY for 3 days every 12 h and with an interval of 6 h during the day for further 2 days before ISR application. ISR was given for 2 days every 8 h (to study tolerability over 2 days) and plasma samples were collected 8 h after the last dose on day 2 or 1 h and 4 h after the dose on day 3 (Figure 2d). All animals, both during the training phase and during ISR treatment, completely consumed their dose within 5 min and no adverse events were recorded. This indicates excellent palatability and tolerability of this dose. The maximal C_PL_ observed was 14.9 ± 5.3 ng/ml (*N* = 4, Table 1) which corresponds to the high end of the therapeutic C_max_ values in humans. After 8 h the C_PL_ fell to values (0.49 ± 0.5 ng/m, *N* = 5, Table 1) close to the lower end of the C_min_ values (0.56–1.4 ng/ml) reported in humans in a recent clinical phase 3 trial (2 × 5 mg/day immediate release formulation [19]) (Figure 2e). From the slope of the curve between 4 h and 8 h an apparent half-life of the C_PL_ decay of about 1.5 h could be estimated (Figure 2f).

To achieve even lower plasma concentrations expected for low-dose treatment in humans ([17,19,20]; SPC Lomir^R^: compendium.ch/mpro/mnr/2604/html/), we treated mice with a single dose of 0.05 mg provided in only 200 µl of SFY (cohort 3, Figure 2g, Supplementary Table S1). During 6 days of training mice were fed 1–2 daily doses of 200 µl of SFY which was consumed within 1 min in 90% of the presentations (3–5 min in the remaining animals, not shown). The C_PL_ was then determined on the next day at 0.5 h, 2 h and 4 h (Figure 2h, i) after a single ISR dose, which was also rapidly consumed (within 1 min in 12/14 mice, 4 min in the remaining two). No likely drug-associated adverse effects were observed in this cohort. One mouse was excluded from the C_PL_ analysis due to unexplained high plasma concentration (4 h time point). At this dose, the highest (2 h: 3.03 ± 0.7 ng/ml, *N* = 5, Table 1) and lowest (4 h: 0.56 ± 0.2 ng/ml, *N* = 4) mean plasma levels closely corresponded to those recently observed for immediate-release formulations in humans (C_max_: 1.86–4.90 ng/ml; C_min_: 0.56–1.40 ng/ml), although elimination in humans occurred with slower kinetics [19].

Notably, a much more frequent blood sampling (i.e. use of a higher number of animals) would be required to capture the C_max_ in our experiments. However, we obtained an estimate for C_max_ by assuming that absorption and elimination kinetics are very similar after administration of 0.1 and 0.05 mg ISR. We normalized the plasma-concentrations in cohorts 2 and 3 to the value at 4 h, which allowed to combine their C_PL_ determined at different time points into one curve. From this we estimated that C_max_ is reached at about 20% higher plasma concentrations than the maximal plasma levels observed at 0.5 h and 2 h (Figure 2j).

Since the 0.1 and 0.05 mg dose range may be suitable for prolonged ISR treatment, we also investigated the level of drug accumulation after treating mice after training (cohort 4, *N* = 3) for 2 days with 0.1 mg ISR in 200 µl SFY at a dose interval of only 4 h (Figure 2k). On day 3 trough plasma concentrations were measured 4 h after the last dose. C_min_ concentrations were 7.42 ± 2.04 ng/ml as compared to 2.36 ± 0.73 ng/ml (Table 1) 4 h after the same dose applied every 8 h (cohort 2). This indicates an accumulation ratio of 3.14-fold which needs to be considered when dosing at a dose interval ≤4 h over several days.

We finally determined the reproducibility of oral dosing at a given dose (cohort 5, *N* = 9) by testing if a target C_PL_ can be obtained at a predetermined time point. Based on our finding that a stable peak C_PL_ can be obtained between 0.5 and 2 h post dose (Figure 2j), we repeated the protocol applied for cohort 2 (0.1 mg ISR every 8 hours over 2 days, Figure 2d) predicting a plasma concentration range similar to the 1 h time point in cohort 2 (Figure 2e). As shown in Figure 2l, M, this was indeed the case. In our study, we determined an elimination half-life after multiple oral dosing of about 1.5 hours. The short half-life is in agreement with the short terminal half-life in humans of about 2.8–3.7 hours after intravenous [17] or oral administration [20].

All mice were included in our safety analysis. The observed adverse events are listed in Supplementary Table S1. No adverse events were observed during the training phase with SFY only. During drug administration, we observed enhanced blood flow upon bleeding both in animals at high but also at lower ISR plasma levels, which is expected from isradipine’s vasodilatory mechanism of action, and were therefore classified as likely drug-related. In the highest dose-group (cohort 1) evidence for increased micturition was suspected in some animals (not shown), a side effect also reported in humans [25]. No other likely-drug related adverse events were observed and tolerability in cohorts 2–5 was excellent as described above (Supplementary Table S1).

## Discussion

Our dose-finding pharmacokinetic study allowed to establish a protocol for the reliable oral application of the CCB ISR in mice. We took advantage of the observation that mice can be trained within days to reliably consume small amounts of SFY (200–500 µl) widely available in food stores at various feeding intervals, even with small amounts (200 µl) given every 4 hours in a multiple-dose study. In addition, Vascal® uno is an extended-release formulation of ISR in which extended-release matrix granules from the capsules may help to stabilize the observed peak plasma level over about 2 hours when ingested as a single dose together with the SFY. We identified the highest dosing regimen (0.5 mg ISR in 500 µl of SFY) as a treatment-limiting dose. This could be attributed to the ISR rather than the SFY because the same volume of SFY was readily accepted during the training phase. We made no attempt to determine whether this was due to impaired palatability by ISR or due to its high dose. The high plasma concentrations measured during the first 4 hours after dosing have also been measured after a single intravenous dose of ISR in humans but are unlikely to be tolerated upon repeated dosing such as in our study (5 doses over 2 days). In contrast, tolerability was excellent for all lower doses. We also observed a clear dose-dependence of plasma levels. Although we did not systematically investigate the dose-dependence, dose reduction from 0.1 mg (multiple dose) to 0.05 mg (single dose) led to only half of the expected plasma concentrations. Nevertheless, our data predict that a dose of 0.05 mg in an adult mouse results in ISR plasma concentrations within the therapeutic range achieved during the oral treatment with recommended therapeutic doses in humans (1–11 ng/ml [17–22]; SPC Lomir^R^: compendium.ch/mpro/mnr/2604/html/). Instead, the 0.1 mg dose leads to higher plasma concentrations known to be tolerated by subjects in pharmacokinetic studies (up to peak plasma concentrations of 14.9–41.8 ng/ml [21,23]). We had no evidence for impaired tolerability of this dose because mice continued to be compliant after switching to the drug-containing SFY after training and showed no obvious adverse effect. This is supported also by preliminary behavioral data. The pharmacokinetic data of cohort 5 were obtained within a study in which the behavior of mice was tested in an elevated plus-maze paradigm [16]. No abnormal behavior was detected at this dose of 0.1 mg (every 8 h over 2 days). This higher tolerable-dose range is relevant to ongoing preclinical studies in mice exploring new indications for DHP CCBs especially if Cav1.3 channels are targeted, which show somewhat lower ISR-sensitivity compared to Cav1.2 [12].

We selected ISR for our study because it has been widely used in preclinical in vitro and in vivo studies. In contrast to some other DHPs it lacks lower affinity targets, such as adenosine transporters (inhibited by nimodipine [26]), inner mitochondrial membrane proteins (interaction with nitrendipine [27]) and binds to Cav1.2 and Cav1.3 with subnanomolar affinity [28]. Moreover, Vascal® uno is to our knowledge the only extended-release formulation of a DHP that can be added to food while preserving its modified-release properties.

A limitation of our study is that we did not test the long-term tolerability of SFY alone so that its suitability for long-term studies with ISR or other drugs in mice is yet unclear. While we found no evidence for alterations in body weight over the training and treatment periods, we cannot exclude effects of long-term SFY-feeding on metabolic parameters that could also interfere with experiments, especially in behavioral studies. At present, we have no experience with other vehicles for administration. Thorough characterization of the tolerability and long-term effects of yogurt-based vehicles on mouse health seem warranted because our approach may also be suitable for the oral administration of other drugs and in other rodents.

In conclusion, we established a protocol for the reliable oral application of the CCB ISR in mice to reproducibly achieve ISR C_PL_ within a wide range previously shown to be well-tolerated in humans. This protocol should facilitate ongoing nonclinical studies in mice exploring new indications for DHP CCBs. It can also be extended to other drugs requiring repeated oral application and in which handling of mice should be avoided.

## Supplementary Material

Supplemental Material

Supplemental Material

## Data Availability

All data of this article are available from the authors upon reasonable request.

## Delarations

No generative AI and AI-assisted technologies have been used in any process of this work.
