# Supplementary material for: Novel protocol for multiple-dose oral administration of the L-type Ca2+ channel blocker isradipine in mice: A dose-finding pharmacokinetic study
Source: Channels (Austin). 2024 Apr 2;18(1):2335469. doi: 10.1080/19336950.2024.2335469 (PMC10989688; doi:10.1080/19336950.2024.2335469)
Supplement: Supplemental Material [file KCHL_A_2335469_SM4643.docx]

**Supplementary material**

**Supplementary Table**

| **Dose** *(mg)* | **Volume** *(µl)* | **Mouse-ID** | **Sex**  *(m/f)* | **Age**  *(months)* | **BW - pre** *(g)* | **BW – post**  *(g)* | **LD interval** *(min)* | **Application duration** *(min)* | **C_PL_**  *(ng/ml)* | **Adverse Events** | |
| --- | --- | --- | --- | --- | --- | --- | --- | --- | --- | --- | --- |
|  |  |  |  |  |  |  |  |  |  | **Event** | *likely drug related* |
| ***Cohort 1*** *(N=13)* | | | | | | | | | | | |
| **0.5** | 500 | 589 | m | 3.8 | 30 | 30 | 74 | 5 | 49.80 | BF | yes |
|  |  | 601 | f | 3.5 | 23 | 24 | 75 | 5 | 69.80 | BF | yes |
|  |  | 585 | f | 4.1 | 25 | 26 | 83 | 5 | 49.00 | BF | yes |
|  |  | 587 | f | 4.1 | 24 | 25 | 241 | 5 | 26.60 | BF | yes |
|  |  | 615^x^ | m | 3.4 | 27 | 26 | 244 | >60, only 25% | 16.70 |  |  |
|  |  | 599 | m | 3.5 | 29 | 26 | 245 | 60 | 59.10 |  |  |
|  |  | 600 | f | 3.5 | 26 | 26 | 247 | 5 | 32.50 |  |  |
|  |  | 603 | m | 3.5 | 28 | 26 | 744 | 60 | 1.13 |  |  |
|  |  | 613 | m | 3.4 | 30 | 27 | 747 | >60, only 75% | 0.46 | AE | no |
|  |  | 617 | f | 3.4 | 22 | 21 | 749 | >60 | 0.52 |  |  |
|  |  | 583 | f | 4.1 | 29 | 28 | 751 | 10 | 0.49 |  |  |
|  |  | 578 | f | 4.4 | 25 | 24 | 756 | >60 | 0.54 |  |  |
|  |  | 582^x^ | m | 4.1 | 33 | 32 | ? | NC | 96.00 | AE | yes |
| ***Cohort 2*** *(N=13)* | | | | | | | | | | | |
| **0.1** | 300 | 640 | f | 3.2 | 21 | 23 | 60 | 5 | 17.10 | BF | yes |
|  |  | 641 | f | 3.2 | 22 | 23 | 63 | 5 | 19.30 | BF | yes |
|  |  | 650 | f | 3.2 | 20 | 21 | 65 | 5 | 16.10 | BF | yes |
|  |  | 647 | m | 3.2 | 27 | 28 | 66 | 5 | 7.30 |  |  |
|  |  | 642 | f | 3.2 | 21 | 22 | 237 | 5 | 1.88 |  |  |
|  |  | 643 | f | 3.2 | 21 | 21 | 240 | 5 | 3.44 |  |  |
|  |  | 651 | f | 3.2 | 23 | 25 | 243 | 5 | 2.11 |  |  |
|  |  | 648 | m | 3.2 | 30 | 30 | 246 | 5 | 1.99 |  |  |
|  |  | 637 | m | 3.2 | 26 | 26 | 467 | 5 | 1.46 |  |  |
|  |  | 652 | f | 3.2 | 23 | 24 | 478 | 5 | 0.27 |  |  |
|  |  | 649 | m | 3.2 | 27 | 28 | 484 | 5 | 0.20 |  |  |
|  |  | 645 | f | 3.2 | 22 | 23 | 485 | 5 | 0.24 |  |  |
|  |  | 632 | f | 3.4 | 22 | 23 | 494 | 5 | 0.27 |  |  |
| ***Cohort 3*** *(N=14)* | | | | | | | | | | | |
| **0.05** | 200 | 2536 | m | 3.1 |  | 27 | 26 | 1 | 2.82 | BF | yes |
|  |  | 660 | f | 3.0 |  | 22 | 29 | 1 | 2.24 | BF | yes |
|  |  | 655 | m | 3.0 |  | 24 | 29 | 1 | 3.11 | BF | yes |
|  |  | 708 | f | 3.1 |  | 21 | 29 | 1 | 3.93 | BF | yes |
|  |  | 654 | m | 3.0 |  | 23 | 119 | 4 | 2.52 |  |  |
|  |  | 659 | f | 3.0 |  | 21 | 119 | 1 | 2.60 |  |  |
|  |  | 658 | f | 3.0 |  | 20 | 119 | 1 | 2.63 |  |  |
|  |  | 699 | m | 3.0 |  | 28 | 119 | 1 | 4.08 | BF | yes |
|  |  | 2535 | m | 3.1 |  | 29 | 120 | 1 | 3.32 |  |  |
|  |  | 656 | m | 3.0 |  | 25 | 239 | 1 | 0.29 |  |  |
|  |  | 2537 | m | 3.1 |  | 26 | 239 | 4 | 0.59 |  |  |
|  |  | 703 | m | 3.0 |  | 28 | 239 | 1 | 0.66 |  |  |
|  |  | 657 | f | 3.0 |  | 20 | 239 | 1 | 0.70 |  |  |
|  |  | 2540^x^ | m | 3.1 |  | 30 | 239 | 1 | 15.95 | BF, AE | yes |
| ***Cohort 4*** *(N=3)* | | | | | | | | | | | |
| **0.1** | 200 | 686 | f | 3.6 |  | 22 | 235 | 1 | 7.35 |  |  |
|  |  | 682 | f | 4.4 |  | 24 | 239 | 1 | 4.95 |  |  |
|  |  | 698 | f | 2.8 |  | 22 | 245 | 1 | 9.95 |  |  |
| ***Cohort 5*** *(N=9)* | | | | | | | | | | | |
| **0.1** | 200 | 725 | f | 3.1 | 21 | 21 | 125 | 1 | 19.65 | BF | yes |
|  |  | 822 | f | 3.4 | 18 | 19 | 126 | 1 | 8.65 |  |  |
|  |  | 727 | f | 3.1 | 21 | 21 | 126 | 1 | 17.90 |  |  |
|  |  | 747 | f | 2.4 | 19 | 20 | 126 | 1 | 26.50 | BF | yes |
|  |  | 827 | f | 3.2 | 20 | 21 | 132 | 1 | 6.65 |  |  |
|  |  | 831 | f | 3.2 | 23 | 23 | 133 | 1 | 6.15 |  |  |
|  |  | 828 | f | 3.2 | 22 | 22 | 135 | 1 | 5.45 |  |  |
|  |  | 829 | f | 3.2 | 23 | 23 | 135 | 1 | 12.15 |  |  |
|  |  | 737 | f | 3.0 | 22 | 22 | 135 | 1 | 23.95 |  |  |

***Supplementary Table 1: Mouse characteristics, drug application, plasma concentration and adverse effects after oral ISR administration.*** *For each cohort we used different dosages (0.5, 0.1, or 0.05 mg) of slow-release ISR mixed in 0.2 and 0.5 ml of SFY. The last dose (LD) interval is the exact time period from the mouse finishing all SFY and bleeding and defined each plasma concentration (C_PL_) time point in our analysis. The application duration was determined for the last dose and indicates the time period from the start of administration until completion of its consumption. ISR plasma concentrations are expressed as C_PL_ (ng/ml). Three animals had to be excluded (marked with "x") for the pharmacokinetic analysis: Mouse 582 (cohort 1) due to its unhealthy condition, reduced drug/yoghurt intake (therefore also referred as not compliant, NC) likely due to an unexplained extremely high ISR C_PL_ of 96 ng/ml and mouse 615 (cohort 1), which only partially consumed the last dose (25%). In addition, mouse 2540 (cohort 3), which showed an abnormally high ISR C_PL_, was also excluded. Animals with any of the following symptoms were labelled as having an adverse event (AE): abnormal appearance with rough hair coat and piloerection, diarrhea, hunched posture, reduced yoghurt intake, reduced mobility. BF indicates increased blood flow during blood collection. Adverse events that were considered "likely drug-related" are indicated. Mouse 615 and 613 (both cohort 1) missed 75% and 25% of their last dose, respectively. X indicates exclusion from C_PL_-analysis but inclusion in safety population. For safety analysis we included all animals.*

**Supplementary methods**

***Drug application procedure:***

- Mice were single housed for one week.
- A 35 mm cell culture dish was glued with double-sided adhesive tape to the bottom of the front part of the cage.
- Bedding material in and around the dish was removed with a brush before treatment onset.
- Training period: Twice or three times a day, at around 8:00, 14:00 and 20:00 hours, 0.2-0.5 ml of drug-free strawberry-flavored yoghurt (SFY, "Fruchtzwerge", Danone, Germany) was presented on the dish, which was glued to the front part of the cage floor (**Fig. 1A, B**). Initially, we opened the mouse cage, placed the SFY, closed the lid and left the mouse as un-disturbed as possible. It took 10 – 60 min for mice to eat the SFY, when it was presented for the first time. Once mice overcame the initial avoidance, they maintained the interest to the SFY and after the training period of 4 - 6 days, the majority of the mice finished the yoghurt within one min. If mice showed no initial interest in the SFY, moving the dish closer to the house usually started their willingness for consumption. Putting little SFY on the fur also helped to stimulate their initial interest.
- Pharmacokinetic study: For the pharmacokinetic study we prepared the ISR-SFY mixture. Therefore, we used a small disposable weighing tray, weighed the yoghurt and mixed in the amount of drug required for the desired concentration using a small spatula. We made sure that the ISR mixture was uniformly spread and not lumpy. The ISR-SFY mixture was always prepared fresh before each treatment and, depending on the protocol, 200 to 500 µl were placed per feeding on the cell culture dish in the front part of the cage. The likelihood that the ISR-SFY mixture was completely consumed was highest at a volume of 200 µl and is therefore recommended. For each mouse treatment took about 3 to 5 min to remove the bedding material, to place the ISR-SFY mixture and to ensure that the mouse has eaten all the yoghurt containing the drug. For the last dose interval, the 0-time point was defined as the time point when the mouse had finished all the yoghurt. The time to plasma collection was determined precisely and used for data analysis.

***Plasma isolation***

Mice were killed by cervical dislocation after sedation with isoflurane. Sufficient blood was acquired by decapitation. Blood was collected using 1ml MiniCollect tubes (greiner bio-one, 450531). These tubes contain the hematocrit sealing agent EDTA to prevent coagulation. For plasma extraction, samples were centrifuged for 10 min at 3,000 rpm (845 × g) and 4°C. Plasma was collected and stored at -20°C until ISR plasma concentrations were determined with a liquid chromatography-tandem mass spectrometry (LC-MS/MS) method.

***Plasma ISR measurements***

ISR concentrations were determined with a liquid chromatography-tandem mass spectrometry (LC-MS/MS) method. An isotopically labeled analogue was used as internal standard (ISR-D3). Water, methanol, acetic acid and acetonitrile (all HPLC grade) were purchased from Honywell (Seelze, Germany). ISR and ISR-D3 were obtained from Toronto Research Chemicals (Toronto, Canada). The LC-MS/MS system consisted of a Waters ACQUITY UPLC H-Class PLUS Bio (Waters, Manchester, UK) and a QTrap 6500+ mass spectrometer (Sciex, Framingham, MA, USA). Chromatographic separations were accomplished on a reversed-phase column (Kinetex 2.6 µm Biphenyl 100 Å, 100 × 2.1 mm, Phenomenex) using a 1-minute linear gradient of 10-98% Methanol in aqueous 0.5% acetic acid solution (v/v). The gradient program was started 1 min after the injection. The flow rate was set to 200 µl/min. The column temperature was held at 50°C. The injection volume was 10 µl. Mass spectrometry detection was performed with electrospray ionization in negative ion mode. Multiple reaction monitoring was carried out using the precursor-to-product ion transitions m/z 370.1 to 250.1 and m/z 370.1 to 119.1 for ISR and m/z 373.1 to 253.1 and m/z 373.1 to 119.1 for ISR-D3. Sample preparation involved a protein precipitation step. Before use, plasma samples were thawed and allowed to equilibrate to room temperature. A 50 µl-aliquot of the sample was mixed with 5 µl of internal standard solution (30 ng/ml) and 95 µl of methanol. The mixture was vortexed and centrifuged at 4,600 × g for 5 min. The obtained supernatant was submitted to LC-MS/MS analysis. Calibration was accomplished by complementing ISR-free mouse plasma with known concentrations of ISR ranging from 0.020 to 100 ng/ml. The limit of quantification was 0.050 ng/ml.

***Pharmacokinetic calculations***

C_PL_ was determined directly from the plasma concentration-time data. Assuming a first-order elimination processes the apparent rate constant for C_PL_ decay and the corresponding half-life during given time points was calculated as follows: and

The accumulation ratio (AR) was calculated from trough plasma concentrations 4 h post dose (cohorts 2 and 4) as:
